# Increase in Ca^2+^-Activated cAMP/PKA Signaling Prevents Hydroxychloroquine-Induced Bradycardia of the Cardiac Pacemaker

**DOI:** 10.3389/fphys.2022.839140

**Published:** 2022-05-11

**Authors:** Sofia Segal, Limor Arbel-Ganon, Savyon Mazgaoker, Moran Davoodi, Yael Yaniv

**Affiliations:** Laboratory of Bioenergetic and Bioelectric Systems, Faculty of Biomedical Engineering, Technion-IIT, Haifa, Israel

**Keywords:** hydroxychloroquine, COVID-19, IBMX, pacemaker, phosphodiesterase inhibition

## Abstract

Bradycardia or tachycardia are known side effects of drugs that limit their clinical use. The heart pacemaker function which control the heart rate under normal conditions is determined by coupled clock system. Thus, interfering with specific clock mechanism will affect other clock mechanisms through changes in interconnected signaling and can lead to rhythm disturbance. However, upregulation of a different clock components can compensate for this change. We focus here on hydroxychloroquine (HCQ), which has been shown effective in treating COVID-19 patients, however its bradycardic side effect limits its clinical use. We aim to decipher the mechanisms underlying the effect of HCQ on pacemaker automaticity, to identify a potential drug that will eliminate the bradycardia. We used isolated rabbit sinoatrial node (SAN) cells, human-induced pluripotent stem cell–derived cardiomyocytes (hiPSC-CMs) and mouse SAN cells residing in SAN tissue. Further, we employed SAN cell computational model to suggest mechanistic insights of the effect of HCQ on pacemaker function. HCQ increased mean spontaneous beat interval and variability in all three models in parallel to slower intracellular kinetics. The computational model suggested that HCQ affects the pacemaker (funny) current (I_f_), L-type Ca^2+^ current (I_Ca,L_), transient outward potassium (I_to_) and due to changes in Ca^2+^ kinetics, the sodium-calcium exchanger current (I_NCX_). Co-application of 3’-isobutylmethylxanthine (IBMX) and HCQ prevented the increase in beat interval and variability in all three experimental models. The HCQ-induced increase in rabbit and mice SAN cell and hiPSC-CM spontaneous beat interval, can be prevented by a phosphodiester inhibitor that restores automaticity due to slower intracellular Ca^2+^ kinetics.

## Introduction

Bradycardia and tachycardia are well known side effects of several drugs and limit their clinical use (Bakhshaliyev et al., 2020; Baracaldo-Santamaría et al., 2021; Moreland-Head et al., 2021). Understanding the mechanisms that attenuate heart rate changes elicited by these drugs is the first step toward eliminating such side effects. Under normal physiological conditions, the sinoatrial node (SAN) controls heart rate and rhythm. Because SAN function is determined by a coupled clock system, downregulating a specific clock mechanism will affect the other clock through interconnecting clock mechanisms. However, upregulation of other clock components may compensate for the downregulated signal and eliminate bradycardia or tachycardia.

Hydroxychloroquine (HCQ) is a drug indicated for the treatment of autoimmune diseases such as rheumatoid arthritis (Lane et al., 2020b) and lupus (James et al., 2007). In the last year, HCQ was repurposed to treat COVID-19, due to its antiviral activity against SARS-CoV (Rolain et al., 2007). Concomitant with the accumulating evidence of its promising clinical benefits, evidence of heart rhythm disturbances, such as bradyarrhythmia, in treated patients has accumulated (Lane et al., 2020a; Borba et al., 2020). Medical charts showed that bradyarrhythmia was more severe when HCQ treatment was combined with other drugs, such as the antibiotic azithromycin, which has a similar bradyarrhythmic side effect. The FDA reacted to these findings by publishing a warning against the use of HCQ or chloroquine outside the hospital setting or in clinical trials (FDA, 2020). A drug that attenuates HCQ-induced bradyarrhythmia can potentially improve its therapeutic efficacy. Identification of such a drug can be facilitated by first investigating the mechanisms underlying the bradycardic effect of HCQ.

HCQ has been shown to directly affect SAN mechanisms. More specifically, 1 µM HCQ was shown to increase beat interval and decrease the pacemaker current I_f_ in isolated guinea pig SAN cells (Capel et al., 2015). At 3 µM and 10 μM, in addition increasing beat interval, HCQ decreased the L-type Ca^2+^ and the rapidly activating potassium currents with a further decrease in I_f_. Because SAN function is determined by a coupled clock system, our first hypothesis is that interfering with the function of these channels will impact other clock mechanisms through changes in Ca^2+^ and Ca^2+^-activated adenylyl cyclase signaling that affect both clocks. Our second hypothesis is that upregulation of phosphorylation activity can compensate for this change.

To test these hypotheses, in this work we investigated the effect of HCQ on pacemaker clock function in single isolated rabbit SAN cells, single human-induced pluripotent stem cell–derived cardiomyocytes (hiPSC-CMs) and mouse SAN cells residing in SAN tissue—three experimental models of spontaneously firing cells. Briefly, in these models, HCQ was shown both experimentally and theoretically, to cause bradycardia which was prevented by co-application of 3′-isobutylmethylxanthine (IBMX).

## Materials and Methods

### Animal Use

Animals were treated in accordance with the Technion Ethics Committee. The experimental protocols were approved by the Animal Care and Use Committee of the Technion (Ethics number: IL-001-01-19 for rabbits and 002-01-19 for mice).

### SAN Tissue and Cell Isolation

SAN cells were extracted from New Zealand white rabbits weighing 2.3–2.7 kg. Rabbits were sedated with ketamine (0.1 ml/kg) and xylazine (0.1 ml/kg) and anesthetized with 200 mg/ml sodium pentobarbital diluted with heparin, administered through an intravenous cannula. The hearts were quickly removed and placed in warm (37°C) Tyrode’s solution containing: 125 mM NaCl, 5.6 mM KCl, 1.2 mM NaH_2_PO_4_, 24 mM NaHCO_3_, 5.6 mM glucose, 21 mM MgCl_2_, and 21.8 mM CaCl_2_, bubbled with 95% O_2_ and 5% CO_2_. The SAN tissue was processed as previously described (Davoodi et al., 2017). Briefly, SAN cells were dispersed by gentle pipetting through a fresh KB solution containing 70 mM L-Glutamic acid, 30 mM KCl, 10 mM KH_2_PO_4_, 10 mM HEPES, 20 mM taurine, 10 mM glucose, 21 mM MgCl_2_, and 0.3 mM EGTA (pH 7.38, with KOH), filtered through a 150 μm mesh.

For experiments involving cultured SAN cells, the cells were diluted in serum-free culture medium mixed with salts and blebbistatin containing 116 mM NaCl, 5.4 mM KCl, 0.8 mM MgCl_2_, 0.9 mM NaH_2_PO_4_, 5.6 mM glucose, 20 mM HEPES, 1.8 mM CaCl_2_, 26 mM NaHCO_3_, 20% M199 (Sigma, supplemented with 5 mM creatine, 2 mM L-carnitine, 5 mM taurine, 0.1% ITS, and 1% penicillin and streptomycin and titrated to pH 7.4 with NaOH, at 37°C) and 25 μM blebbistatin. The suspended cells were seeded and incubated (37°C, 90% humidity, 5% CO_2_, Galaxy 170R, Eppendorf) as previously described (Segal et al., 2019). After 1 h the medium was replaced with serum-enriched culture medium containing 116 mM NaCl, 5.4 mM KCl, 0.8 mM MgCl_2_, 0.9 mM NaH_2_PO_4_, 5.6 mM glucose, 20 mM HEPES, 1.8 mM CaCl_2_, 26 mM NaHCO_3_), 20% M199 (Sigma, supplemented with 5 mM creatine, 2 mM L-carnitine, 5 mM taurine, 0.1% insulin-transferrin-selenium-X, 4% fetal bovine serum, 2% horse serum, and 1% penicillin and streptomycin and titrated to pH 7.4 with NaOH at 37°C) and 25 μM blebbistatin.

For experiments using whole SAN tissue, adult (12–14 weeks, 25–30 g) male C57BL mice were anesthetized with sodium pentobarbital (50 mg/kg, intraperitoneal (i.p.)) diluted with heparin. The hearts were quickly removed and placed in a 37°C Tyrode’s solution containing 140 mM NaCl, 1 mM MgCl_2_, 5.4 mM KCl, 1.8 mM CaCl_2_, 5 mM HEPES, and 5 mM glucose, pH 7.4 (titrated with NaOH). The SAN tissue was isolated from the intact heart as previously described (Neco et al., 2012; Wang et al., 2017). Briefly, the SAN and the surrounding atrial tissue were dissected and pinned down in custom-made silicone-covered optical chambers, bathed in the above SAN tissue Tyrode’s solution.

### Human-Induced Pluripotent Stem Cell Generation and Cardiomyocyte Differentiation

Human-induced pluripotent stem cells (hiPSCs; clone 24.5) were generated from human dermal fibroblasts using Sendai virus CytoTune-iPS 2.0 and Sendai Reprogramming Kit, #A16517 (Thermo Fisher, Waltham, MA, United States) for the transfection of Yamanaka’s four factors: 4 Oct, Klf4, c-Myc, Sox2, as previously described (Takahashi and Yamanaka, 2006; Novak et al., 2015). Before biopsy collection, the 42-year-old healthy female donor signed a consent form according to approval #3116 by the Helsinki Committee for experiments on human subjects at Rambam Health Care Campus, Haifa, Israel. hiPSCs were differentiated into cardiomyocytes (hiPSC-CMs) by modulating Wnt/β-catenin signaling, as previously described (Lian et al., 2013).

### Ca^2+^ Imaging

Ca^2+^ fluorescence was imaged using a LSM880 confocal microscope, equipped with a 40x/1.2 water immersion lens, at 37 ± 0.5°C, as described before (Davoodi et al., 2017). Briefly, cells were excited with a 488 nm argon laser and fluorescence emission was collected with LP 505 nm. Images were acquired from spontaneously beating SAN cells in line scan mode (1.22 ms per scan; pixel size, 0.01 μm), from the long axes of pacemaker cells, from spontaneously beating hiPSC-CMs and from SAN cells within the primary spontaneously beating SAN tissue, where each cell was imaged before (control) and after drug administration.

Rabbit SAN cells were loaded with 5 μM Fluo-4 AM (Thermo Fisher, 30 μmol/L) for 20 min in the dark, at room temperature, and washed with 37°C HEPES buffer containing:140 mM NaCl, 5.4 mM KCl, 5 mM HEPES, 10 mM glucose, 22 mM MgCl_2_, 21 mM CaCl_2_ (pH 7.4, with NaOH). Experiments were performed on fresh cells less than 6 h after isolation or on cultured cells after washout of blebbistatin. hiPSC-CMs were loaded with 2.5 μM Flou-4 AM for 20 min in the dark, at room temperature, and then washed with Tyrode’s solution (at 37°C) containing 140 mM NaCl, 5.4 mM KCl, 10 mM HEPES, 2 mM Na-pyruvate, 10 mM glucose, 1 mM MgCl_2_ and 2 mM CaCl_2_ (pH 7.4, with NaOH). Intact SAN preparations were loaded with Fluo-4-AM (30 μmol/L) for 1 h at 37°C and place on a shaker at 60 RPM. The tissues were washed twice with 37°C Tyrode’s solution containing 140 mM NaCl, 1 mM MgCl_2_, 5.4 mM KCl, 1.8 mM CaCl_2_, 5 mM HEPES, and 5 mM glucose, pH 7.4 (titrated with NaOH).

Ca^2+^ analysis was performed using a modified version of Sparkalizer (Davoodi et al., 2017). The signal (F) was normalized by the minimal value between beats (F_0_). Ca^2+^ transients were semi-automatically detected and Ca^2+^ sparks were manually marked. Beat interval was calculated as the mean beat interval of each cell, and the variability was calculated as its standard deviation (SD). Relaxation time (50% and 90%) and spark parameters were automatically calculated by the software as we have previously described (Davoodi et al., 2017).

### Computational Model

To predict the mechanisms underlying the effect of HCQ on the spontaneous beat rate, simulations were performed using our previously published rabbit SAN cell model (Behar et al., 2016). Beat interval was calculated as the average distance between membrane potential peaks after model stabilization. Numerical integration was performed using the MATLAB (The MathWorks, Inc. Natick, MA, United States) ode15s stiff solver, and the model simulations were run for 100 s to ensure that steady state was attained. Computation was performed on an Intel(R) Core (TM) i7–4790 CPU @ 3.60 GHz machine with 8 GB RAM. The source code of the numerical model is available at: http://bioelectric-bioenergetic-lab.net.technion.ac.il.

### Drugs

HCQ and IBMX were purchased from Sigma-Aldrich.

### Statistical Analysis

Data are presented as mean ± standard error of the means (SEM), and compared using paired or unpaired student’s t-test or one-way analysis of variance (ANOVA). Differences were considered statistically significant at *p* < 0.05. To determine whether the shapes of two distributions were the same, the Kolmogorov-Smirnov two-sample test was applied to the standard scores (z-scores) from each sample. The standard scores were obtained by subtracting the sample-specific mean and dividing the result by the sample-specific standard deviation for each group.

## Results

### The Effects of HCQ on the Spontaneous Beat Interval and Rhythm of Isolated Rabbit SAN Cells

**FIGURE 1:**
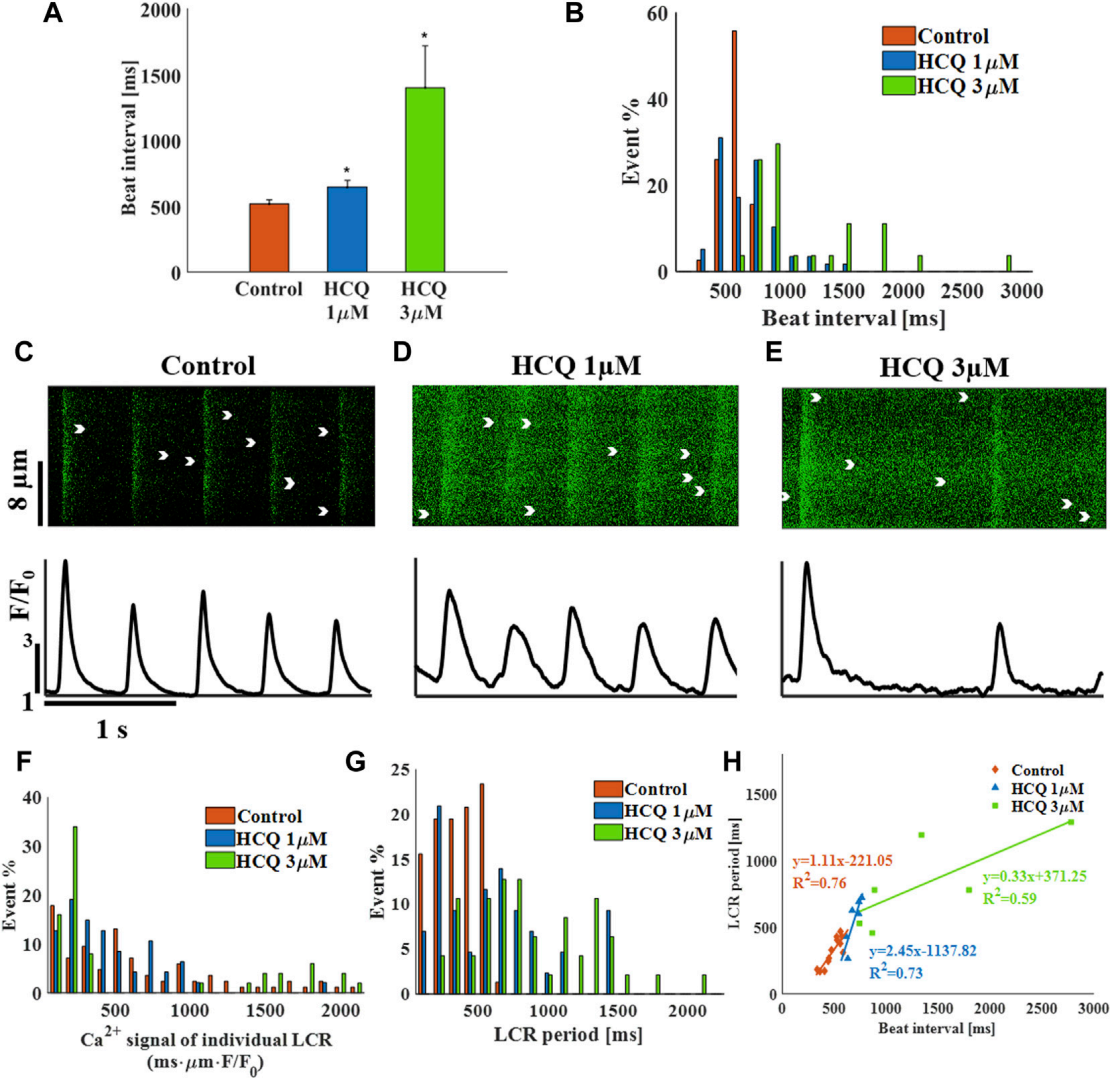
The effect of hydroxychloroquine (HCQ) on single rabbit sinoatrial node cells. **(A)** Mean beat interval and **(B)** beat interval distribution in control (*n* = 7) and in rabbit sinoatrial cells treated with 1 μM (*n* = 7) and 3 μM (*n* = 6) HCQ. Representative Ca^2+^ transients in **(C)** control cells, and in cells treated with **(D)** 1 μM or **(E)** 3 μM HCQ. The distribution of **(F)** the Ca^2+^ signal of individual LCRs and **(G)** the LCR period in control cells and cells treated with 1 μM or 3 μM HCQ. **(H)** The correlations between LCR period and beat interval in control cells and in cells treated with 1 μM or 3 μM HCQ. *p < 0.05 vs. control.

We first tested the effect of HCQ on the spontaneous beating interval and rhythm of isolated single rabbit SAN cells. At 1 μM and 3 μM, HCQ increased beat interval; 24.7 ± 7.8% and 170.8 ± 22.7%, respectively, compared to control (Figure 1A). Further, both doses increased the beat interval variability, as quantified by the SD (Supplementary Table S1) and each increased the beat interval distribution compared to control (Figure 1B). Next, we investigated whether the increase in beat interval is associated with changes in the coupled clock function. As illustrated by the representative Ca^2+^ transients (Figures 1C–E) and Supplementary Table S1, we found that 1 µM and 3 μM HCQ increased the 50% and 90% Ca^2+^ transient relaxation times. Notably, the changes in sarcoplasmic reticulum (SR) local Ca^2+^ release (LCR) parameters (including its period) reflect alterations in both membrane currents and the Ca^2+^ clock machinery (Yaniv et al., 2014). Specifically, increased in HCQ concentration caused a progressive decrease in LCR length and Ca^2+^ signal magnitude of individual LCRs (Supplementary Table S1). Figure 1F depicts that the distribution of Ca^2+^ signal magnitude of individual LCRs does not change compared to control. Both HCQ concentrations increased LCR period (Supplementary Table S1) along with increased distribution compared to control (Figure 1G). In addition, the slope and correlation coefficient between beating interval and LCR period was altered by HCQ (Figure 1H); whereas at 1 μM, a correlation existed (changed from 0.87 in control to 0.86) with a different slope as compared to the control, at 3 μM, low correlation was found between the two (0.76).

**FIGURE 2:**
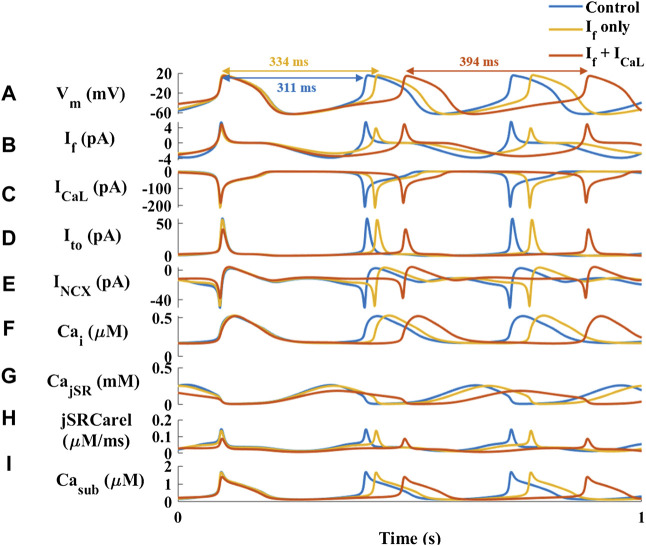
Major currents and Ca^2+^ cycling in simulated rabbit sinoatrial node cells in response to 1 µM hydroxychloroquine. Coupled-clock function of control (blue) the response to only decrease in HCN (funny) current (I_f_) (red) and decrease in I_f_ and L-type Ca^2+^ current (I_CaL_) (yellow). **(A)** Membrane voltage (V_m_) and representative beat intervals, **(B)** I_f_, **(C)** I_CaL_, **(D)** transient potassium current (I_t0_), **(E)** Na^+^-Ca^2+^ exchanger current (I_NCX_), **(F)** intracellular Ca^2+^ concentration (Ca_i_), **(G)** Ca^2+^ concentration in the junctional SR compartment (Ca_jSR_), **(H)** flux of Ca^2+^ exiting the SR (j_SRCarel_) and **(I)** Ca^2+^ concentration in the subspace (Ca_sub_).

To explore the mechanisms underlying HCQ-induced bradycardia, the effect of 1 μM HCQ on a single SAN cell was simulated using our previously published computational model (Behar et al., 2016). Based on the finding that HCQ (1–10 μM) inhibits I_f_ in guinea pig SAN cells (Capel et al., 2015), we firstly focused on this key pacemaker current. Decreasing I_f_ density by 35% (the maximal decrease in I_f_ observed at 10 μM HCQ in guinea pig SAN cells) led to only a 7% increase in beat interval (Figures 2A,B), which is smaller than the 25% increase observed experimentally (Figure 1A). Additionally, a 70% decrease in I_f_ density (the maximal decrease that still produces stable results by the model) increased the beat interval by 20%, which agrees with the effect found experimentally (Supplementary Figure S1). However, no changes in L-type Ca^2+^ current (I_Ca,L_) were obtained, in contrast to experimental results (Capel et al., 2015). Because the model considers both direct and indirect (through changes in Ca^2+^ influx rate and cAMP/PKA levels) effects of I_f_ on pacemaker function, we concluded that a direct effect of HCQ solely on I_f_ cannot explain the effect of HCQ on the beat interval.

The guinea pig experiments further showed that 10 μM HCQ (3 μM were not tested) attenuated the I_Ca,L_ and potassium current (I_Kr_) densities by 12% and 35%, respectively (Capel et al., 2015). Reduction of only the I_Ca,L_ density by 10% increased the beat interval by 16%, which is smaller than the increase observed here experimentally. Moreover, no change was observed in I_f_, in contrast to the experimental results (Capel et al., 2015) (Supplementary Figure S2). Next, we tested the effect of reducing both I_Ca,L_ and I_f_ (as observed in experiments with 1 μM HCQ (Capel et al., 2015)) densities by 10% (Figure 2C). The resulting 21% increase in beat interval agreed with the effect found experimentally (Figure 1A). In contrast to the experimental observations, adding a direct effect on I_Kr_ shortened the beat interval. Under both simulated conditions (i.e., a direct effect on I_f_ with/without an effect on I_Ca,L_) there was no effect on I_Kr_. In contrast, due to an indirect simulated effect of HCQ on these current densities and kinetics, the transient outward potassium current (I_to_) and the sodium-calcium exchanger current (I_NCX_) were decreased by 30% (Figures 2D,E). Additionally, the model was used to explore Ca^2+^ kinetics in different intracellular compartments. 1 μM HCQ did not affect cytosolic Ca^2+^ (Figure 2F). However, the model also predicted reductions in junctional sarcoplasmic reticulum (JSR) Ca^2+^ (Figure 2G) and SR Ca^2+^ release (Figure 2H), resulting in decreased Ca^2+^ concentration in the subspace (Figure 2I), and smaller Ca^2+^ release from the SR.

Because the model predicts indirect changes in I_to_ and I_NCX_, we used the computational model to test the direct effect of reduction in the conductance of these currents. Reduction of I_to_ by 80% decreased the beat interval by 3% (Supplementary Figure S3). Thus, its effect on spontaneous beating rate is negligible. Reduction of I_NCX_ by 40% increased the beat interval, in agreement with the effect found experimentally (Figure 1A). However, no change in I_f_ was documented, in contrast to the experimental results (Capel et al., 2015).

### The Effects of HCQ on the Beat Interval and Rhythm of hiPSC-CMs

**FIGURE 3:**
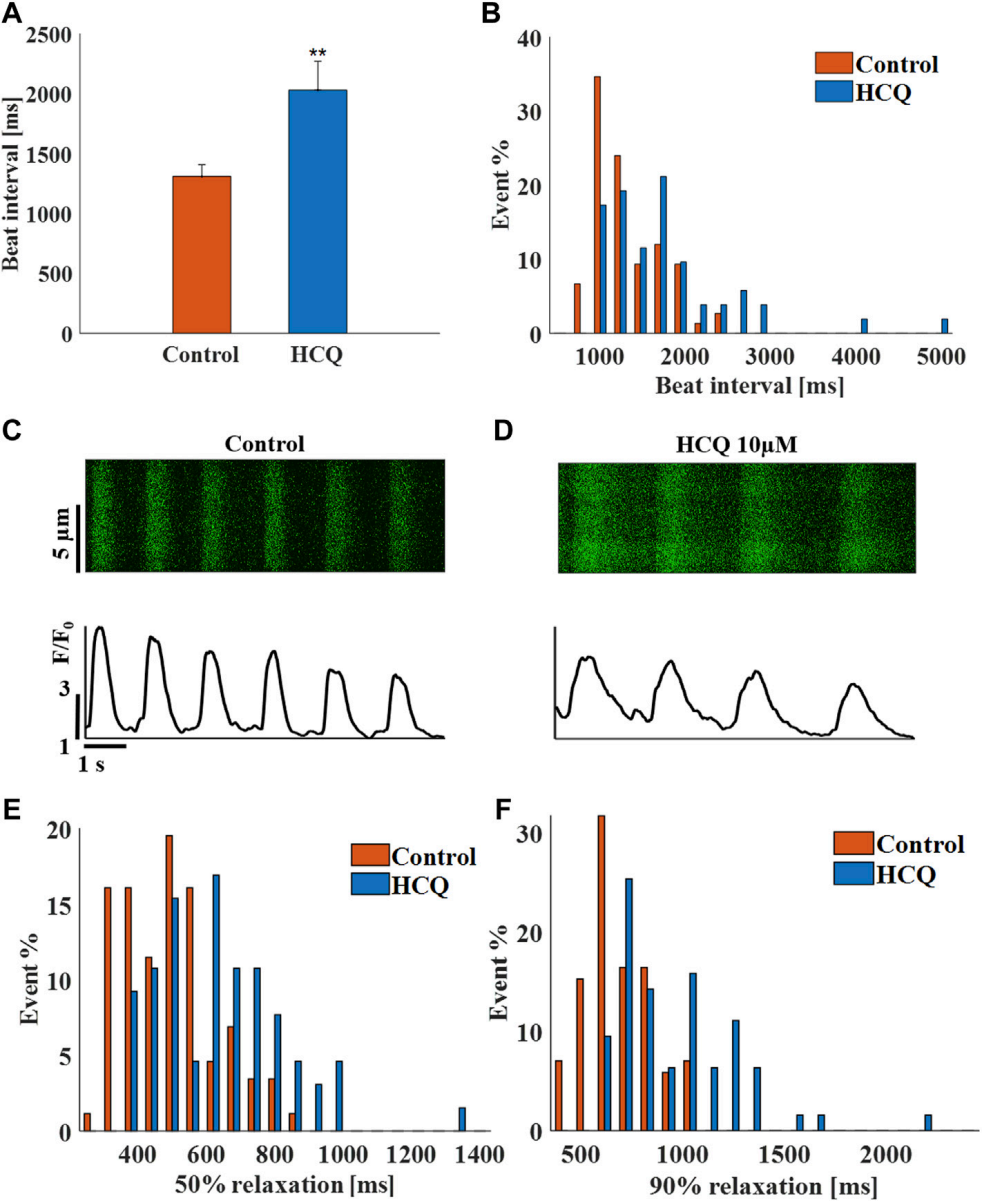
The effect of hydroxychloroquine (HCQ) on human induced pluripotent stem cell-derived cardiomyocytes (iPSC-CM). **(A)** Mean beating interval and **(B)** beat interval distribution of control and human induced pluripotent stem cell-derived cardiomyocytes (iPSC-CM) treated with 10 μM HCQ (*n* = 18). Representative Ca^2+^ transients in **(C)** control and in **(D)** 10 μM HCQ-treated iPSC-CMs. The distribution of **(E)** 50% and **(F)** 90% Ca^2+^ transient relaxation time in control and in 10 μM HCQ-treated iPSC-CMs. **p < 0.01 vs. control.

To demonstrate that the effect of HCQ on automaticity is not unique to rabbit SAN cells, experiments were repeated using spontaneously beating hiPSC-CMs. Whereas 1 μM (−5.2 ± 13.5% compared to control, *n* = 8) and 3 μM (5.4 ± 22.4% compared to control, *n* = 8) HCQ did not affect beat interval, 10 μM HCQ increased beat interval by 55 ± 11.8% (Figure 3A), as well as beat interval variability (Supplementary Table S2) and beat interval distribution (Figure 3B). As illustrated by the representative examples, 10 μM HCQ increased the 50% and 90% Ca^2+^ transient relaxation times (Figures 3C–E; Supplementary Table S2). Additionally, 50% and 90% Ca^2+^ transient relaxation times and increased their distribution by 10 μM HCQ (Figures 3E,F).

### The Effects of HCQ on the Beat Interval and Rhythm of Mouse SAN Tissue

**FIGURE 4:**
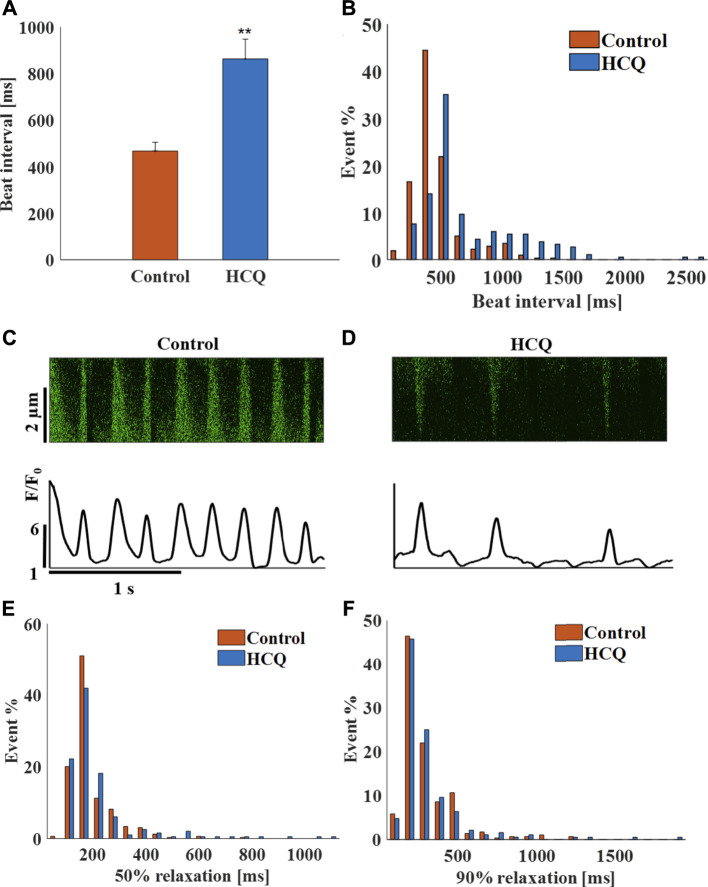
The effect of hydroxychloroquine (HCQ) on mice sinoatrial (SAN) cells residing in the SAN. **(A)** Mean beating interval and **(B)** beat interval distribution in control and in 10 μM HCQ-treated SAN (*n* = 23). Representative example of Ca^2+^ transients in **(C)** control and in **(D)** 10 μM HCQ-treated SANs. The distribution of **(E)** 50% and **(F)** 90% Ca^2+^ transient relaxation time in control or 10 μM HCQ-treated SAN. **p < 0.01 vs. control.

Mouse SAN tissue were treated with HCQ to determine its effect at the tissue level. As observed for the hiPSC-CMs, whereas 1 μM (5 ± 11.2% compared to control, *n* = 17) and 3 μM (4 ± 14.5% compared to control, *n* = 11) HCQ did not affect beating interval, 10 μM HCQ increased beat interval by 84.3 ± 10% (Figure 4A). Further, 10 µM HCQ increased beat interval variability (Supplementary Table S3) and its distribution (Figure 4B). Figures 4C,D show representative Ca^2+^ transients in control and in 10 μM HCQ-treated SANs; the 50% and 90% Ca^2+^ transient average relaxation times and the distribution were comparable to control (Figures 4E,F).

### Preventing the Bradycardic Effect of HCQ

In an attempt to alleviate the bradycardic effect of HCQ, we searched for an FDA-approved drug that can prevent this effect, by restoring Ca^2+^ kinetics as well as I_f_ and I_Ca,L_ conductance (Capel et al., 2015). As a proof-of-concept, IBMX decreased SAN cell and tissue beat interval by increasing Ca^2+^ -dependent phosphorylation activity, and decreased beat interval in pacemaker cell and tissue in response to an increased beat interval associated with aging (Yaniv et al., 2016) or genetic mutation such as catecholaminergic polymorphic ventricular tachycardia (Arbel-Ganon et al., 2020). We choose an IBMX concentration that will reverse the beating interval shortening when concomitantly delivered with HCQ based on previous reports (Vinogradova et al., 2008).

**FIGURE 5:**
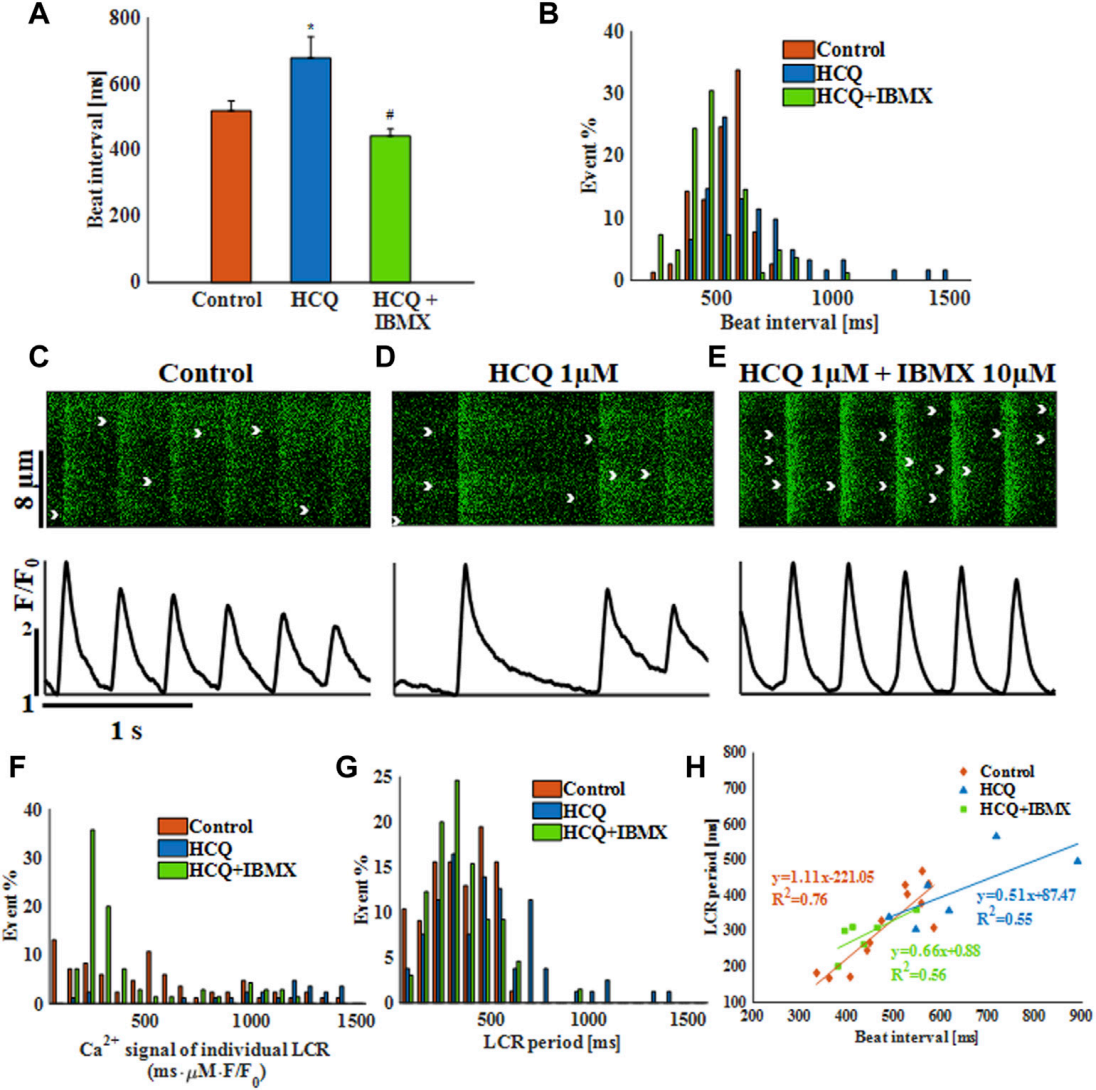
The effect of hydroxychloroquine (HCQ) with 3′-isobutylmethylxanthine (IBMX) on single rabbit sinoatrial node cells. **(A)** Mean beat interval and **(B)** beating interval distribution of control and of 1 μM HCQ- (*n* = 7) or 1 μM HCQ + 10 μM IBMX-treated single rabbit sinoatrial node (SAN) cells (*n* = 7). Representative example of Ca^2+^ transients in **(C)** control and **(D)** 1 μM HCQ- or **(E)** 1 μM HCQ +10 μM IBMX-treated SAN cells. The distribution of **(F)** Ca^2+^ signal of individual LCRs, and **(G)** LCR period in control and in 1 μM HCQ- or 1 μM HCQ and 10 μM IBMX-treated SAN cells. **(H)** The correlations between LCR period and beat interval in control and in 1 μM HCQ- or 1 μM HCQ and 10 μM IBMX-treated SAN cells. *p < 0.05 vs. baseline, ^#^ *p* < 0.05 vs. HCQ 1 μM.

Figure 5A shows that addition of 10 μM IBMX to 1 μM HCQ prevented the increase in beat interval induced by the latter (14.6 ± 10% compared to control) and prevented the increased beat interval variability (Supplementary Table S4) and increased beat interval distribution compared to pre-HCQ levels (Figure 5B). As visualized by the representative examples (Figures 5C–E), in the presence of IBMX, 50% and 90% Ca^2+^ transient relaxation times were comparable to pre-HCQ levels (Supplementary Table S4). Further, IBMX + HCQ restored average LCR length and average and distribution of Ca^2+^ signal of individual LCRs to pre-HCQ levels (Figure 5F; Supplementary Table S4). In the presence of HCQ and IBMX, the average (Supplementary Table S4) and the distribution of the LCR period (seen in Figure 5G) were similar to pre-HCQ levels. In addition, HCQ + IBMX partially restored the correlation (x = 0.76) between beat interval and LCR period, in contrast to the lack of correlation found in HCQ-treated cells (Figure 5H). We also tested the effect of application of 10 μM IBMX to 3 and 10 μM HCQ. Supplementary Figure S5 shows a shift in the dose response curve in response of HCQ in the presence of IBMX. Moreover, in the presence of 10 μM H-89 (PKA inhibitor) the effect of IBMX on restoration of HCQ associated bradycardia was lower.

**FIGURE 6:**
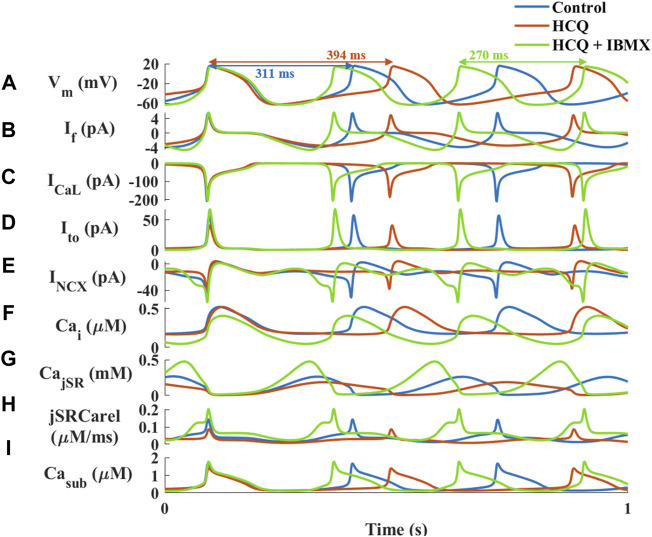
Major currents and Ca^2+^ cycling in simulated rabbit sinoatrial node cells in response to 1 μM hydroxychloroquine (HCQ) and 10 μM isobutylmethylxanthine (IBMX) treatment. Coupled-clock function of control (blue), and of HCQ- (yellow) or HCQ + IBMX-treated (green) rabbit sinoatrial node cells. **(A)** Membrane voltage (V_m_) and representative beat intervals, **(B)** I_f_, **(C)** I_CaL_, **(D)** transient potassium current (I_t0_), **(E)** Na^+^-Ca^2+^ exchanger current (I_NCX_), **(F)** intracellular Ca^2+^ concentration (Ca_i_), **(G)** Ca^2+^ concentration in the junctional SR compartment (Ca_jSR_), **(H)** flux of Ca^2+^ exiting the SR (j_SRCarel_) and **(I)** Ca^2+^ concentration in the subspace (Ca_sub_).

Next, computational modeling was conducted to identify putative mechanisms responsible for the ability of IBMX to prevent the effect of HCQ on the pacemaker. The model suggests that in response to IBMX and HCQ, the beat interval decreased by 15% compared to control, in contrast to the 21% increase (measured experimentally) caused by HCQ alone (Figure 6A). Addition of IBMX to HCQ prevented the decrease in I_f_ (Figure 6B, 8% reduction), I_Ca,L_ (Figure 6C, 7% reduction), I_to_ (Figure 6D, 6% increase) and I_NCX_ (Figure 6E, 4% increase). As found experimentally, adding IBMX restored Ca^2+^ kinetics (Figure 6F) and JSR Ca^2+^ (Figure 6G) and Ca^2+^ release from the SR (Figure 6H). Consequently, Ca^2+^ in the subspace returned to its pre-HCQ levels (Figure 6I).

**FIGURE 7:**
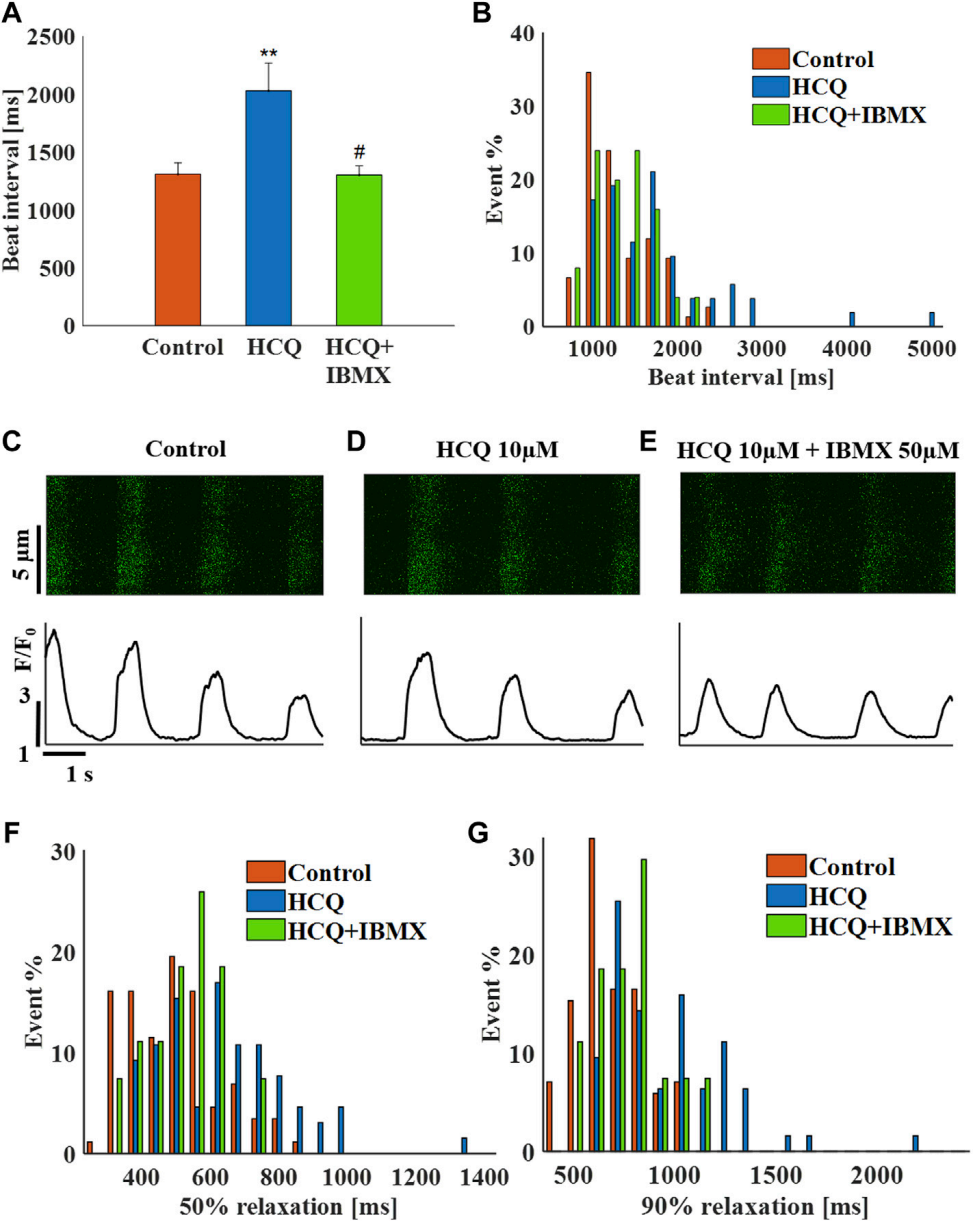
The effect of hydroxychloroquine (HCQ) with 3′-isobutylmethylxanthine (IBMX) on human induced pluripotent stem cell–derived cardiomyocytes. **(A)** Mean beat interval and **(B)** beating interval distribution of control and of 10 μM HCQ- (*n* = 18) or 10 μM HCQ and 50 μM IBMX-treated (*n* = 6) human induced pluripotent stem cell–derived cardiomyocytes (hiPSC-CMs). Representative example of Ca^2+^ transients in **(C)** control and in **(D)** 10 μM HCQ- or **(E)** 10 μM HCQ and 50 μM IBMX-treated hiPSC-CMs. The distribution of **(F)** 50% and **(G)** 90% Ca^2+^ transient relaxation times in control and in 10 μM HCQ- or 10 μM HCQ and 50 μM IBMX-treated hiPSC-CMs. **p < 0.01 vs. baseline, ^#^ *p* < 0.05 vs. 10 μM HCQ.

In agreement with the experiments in the previous models, treatment of hiPSC-CMs with 50 μM IBMX +10 μM HCQ prevented the increase in beat interval (−1 ± 6% compared to control; Figure 7A), as well as the increase in beat interval variability (Supplementary Table S2) and beat interval scattering (Figure 7B). As visualized by the representative Ca^2+^ transients (Figures 7C–E) and quantified in Supplementary Table S2, 50% and 90% Ca^2+^ transient relaxation times and distributions (Figures 7F,G) were comparable to pre-HCQ levels. Note that a 10 μM IBMX and 10 μM HCQ did not reverse the increase in beat interval by 10 μM HCQ alone (5 ± 4%, *n* = 5).

**FIGURE 8:**
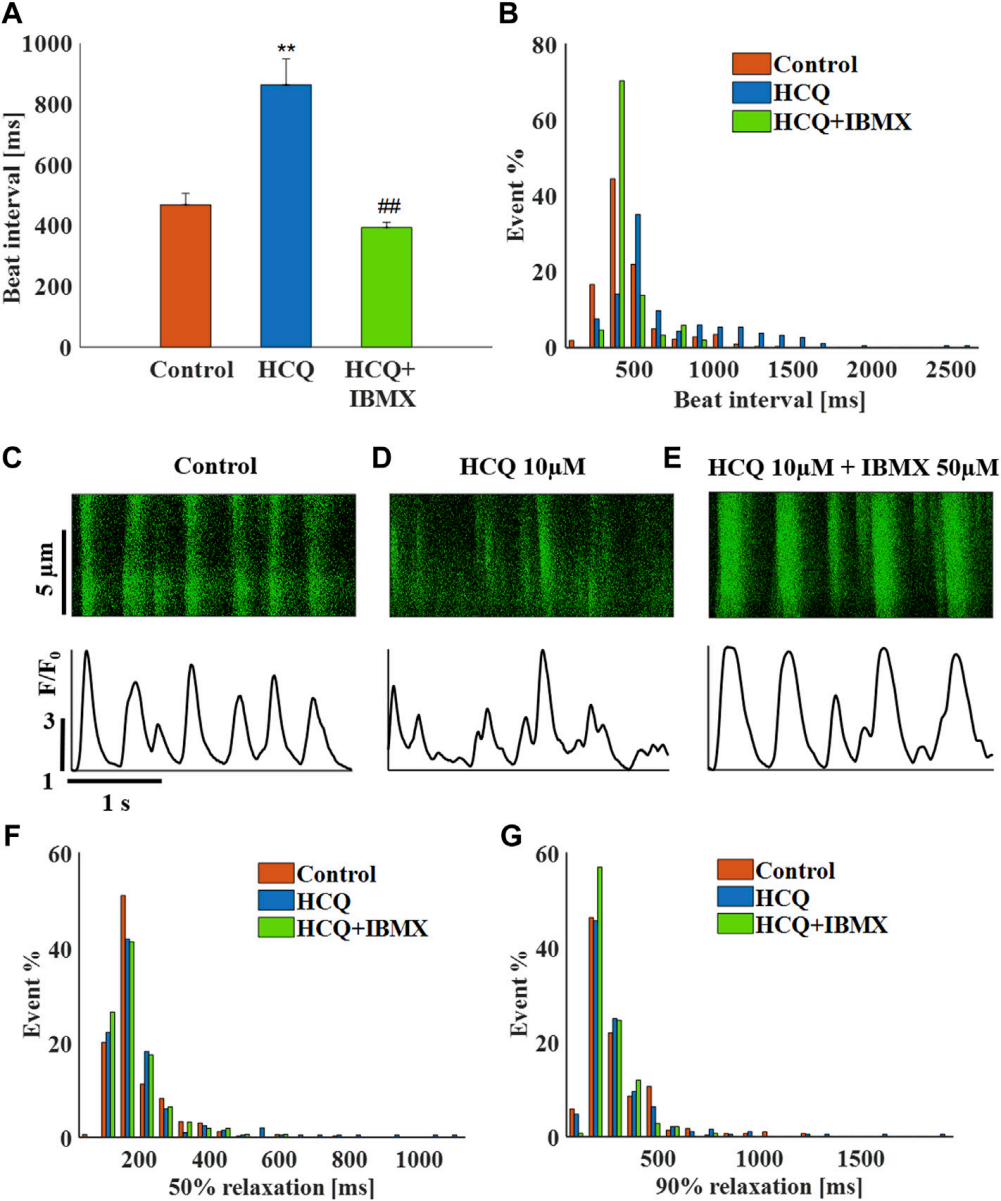
The effect of hydroxychloroquine (HCQ) with 3′-isobutylmethylxanthine (IBMX) on mouse SAN cells residing in the sinoatrial node. **(A)** Mean beat interval and **(B)** beating interval distribution of control and of 10 μM HCQ (*n* = 23) or 10 μM HCQ and 50 μM IBMX-treated mouse sinoatrial nodes (SANs) (*n* = 13). Representative example of Ca^2+^ transients in **(C)** control and in **(D)** 10 μM HCQ- or **(E)** 10 μM HCQ and 50 μM IBMX-treated SANs. The distribution of **(F)** 50% and **(G)** 90% Ca^2+^ transient relaxation times in control and in 10 μM HCQ- or 10 μM HCQ and 50 μM IBMX-treated SANs. **p < 0.01 vs. baseline, ^##^ *p* < 0.01 vs. 10 μM HCQ.

Finally, in SAN tissue, 50 μM IBMX and 10 μM HCQ prevented the increase in beat interval induced by HCQ alone (−16 ± 6% compared to control). The combined treatment also prevented the increase in beat interval variability (Supplementary Table S3) and beat interval scattering (Figure 8B). As visualized by the representative Ca^2+^ transients (Figures 8C–E) and quantified in Supplementary Table S3, in the presence of IBMX and HCQ, 50% and 90% Ca^2+^ transient relaxation times were comparable to pre-HCQ levels including their distribution (Figures 8F,G). Note that a 10 μM IBMX plus 10 μM HCQ regimen did not reverse the increase in beat interval by 10 μM HCQ alone (10 ± 8%, *n* = 10).

## Discussion

The present study tested the effect of HCQ on the function of single isolated rabbit SAN cells, hiPSC-CMs and mice SAN cells residing in the SAN tissue. The findings supported the main hypothesis which suggested that changes in the coupled clock system underlie HCQ-induced bradycardia. In all three models, HCQ was shown to affect the cell beat interval and rhythm, which was prevented by adding IBMX to HCQ. The computational model of the single rabbit SAN cells predicted the mechanisms driving these effects.

Our first main finding was that the known effect of HCQ on beat interval is mediated through changes in coupled clock function. Because LCR parameters and specifically LCR period, correlate with the degree of clock coupling (Yaniv et al., 2013; Yaniv et al., 2014; Lyashkov et al., 2018), manipulating these parameters caused altered coupled clock function. As shown in single rabbit SAN cells, HCQ increased LCR period and beat interval. Increased beat interval caused by decreased ionic currents density and/or internal pacemaker mechanisms lead to a net reduction Ca^2+^ influx per time unit, directly via changes in I_Ca,L_ and/or indirectly due to delayed activation of I_Ca,L_. In turn, this leads to reduced Ca^2+^ available for pumping into the SR, prolongation of the LCR period and a shift of Ca^2+^ activation of I_NCX_ which occurs during diastolic depolarization. Changes in the diastolic depolarization phase feedbacks on ionic current kinetics leading to further increases in beating interval. Although LCR was only measured in single rabbit SAN cells, prolonged time to 90% relaxation, previously associated with reduced SR load and prolonged LCR period (Vinogradova et al., 2010), was demonstrated in hiPSC-CMs and mice SAN cells. residing in SAN tissues. Thus, all three models show reduced coupling between the pacemaker clocks in response to HCQ.

The computational model suggested that reduction in membrane ion currents initiated the reduced pacemaker function in response to HCQ. An earlier work has shown that I_f_ is reduced in response to HCQ even at a low concentration (1 μM), and reduction in I_Ca,L_ and I_kr_ were documented at 10 μM (Capel et al., 2015). Our computational model predicted that at 1 μM, a direct effect on I_f_ cannot fully account for the increase in beat interval. Specifically, in most species, I_f_ was activated at voltages more negative than end diastolic voltages (between −55 mVs and −60 mVs), with time constants of seconds, which is much too slow to make a significant impact alone on beat interval (Yaniv et al., 2012). A direct effect on I_Ca,L_ and I_f_ can explain the increase in beat interval, but a parallel reduction in I_k_ reduced this effect below the experimentally measured level. The model also suggested that although I_Ca,L_ and I_f_ are directly affected by HCQ, the increase in beat interval is also mediated through an indirect decrease of I_to_ and I_NCX_. We attempted to determine whether other coupled clock mechanisms in the model are directly affected by HCQ along with I_f_. Isolated reduction in I_NCX_, I_to_ or I_Ca,L_ cannot explain these results. A reduction in SERCA pump activity led to increased beat interval similar to the effect of HCQ experimentally, but could not explain the reduction in I_Ca,L_ documented at higher doses of HCQ and the changes in Ca^2+^ dynamics. Based on the computational model, HCQ has a direct effect on the “membrane clock,” which leads to an initial increase in beat interval. Increase in beat interval decreases the cross-talk between the L-type current and SR, which subsequently reduces and delays LCRs. Reduced LCRs further disactivate the “membrane clock” through changes in I_NCX_. In parallel, reduction in Ca^2+^-activated AC-cAMP/PKA signaling disactivates both membrane and Ca^2+^ signaling. Thus, the direct effect on the membrane clock leads to an indirect effect on the “Ca^2+^ clock” through clock coupling that further affects the membrane clock. Future experiments on permeabilized pacemaker cells will be required to show that the SERCA pump is not directly affected by HCQ and to verify that I_Ca,L_ and I_f_ are indeed decreased in rabbit SAN cells, as was shown in other SAN cell types.

The second main fining is that concomitant to the increase in beat interval caused by HCQ, increased beat interval variability was documented in all three experimental models. This finding fits well with the effect of other drugs that reduce the coupled clock function in isolated single SAN cells and in SAN cells residing in the SAN (Rocchetti et al., 2000; Yaniv et al., 2014; Yaniv et al., 2016). Increased beat interval variability is associated with arrythmia and thus poses a potential risk for patients for example with atrial fibrillation (Hooks et al., 2020).

The third and main finding is that addition of IBMX prevented the bradycardic effect of HCQ and its effect on increased beat interval variability in all three experimental models. Although some patients can benefit from the bradycardic effect of HCQ, it can put others at risk, specifically COVID-19 patients suffering from reduced breathing rate or patients with preexisting arrhythmia. The computational model suggests that IBMX does not counteract the direct effects of HCQ on membrane currents, but, rather, counteracts the indirect effects of the drug. Addition of IBMX increases cAMP/PKA activity, which enhances SERCA activity and increases the availability Ca^2+^ to be pumped into the SR. Thus, shifting Ca^2+^ activation of I_NCX_ to an earlier point during diastolic depolarization, increases the influx of Ca^2+^ through L-type channels due to enhanced L-type-SR crosstalk. In parallel, IBMX increases cAMP/PKA activity which enhances I_f_. Reversed I_f_, I_Ca,L_, and I_NCX_ through changes in Ca^2+^ decrease the beat interval until its return to baseline level.

The presented observations have direct implications for COVID-19 patients and management of autoimmune diseases, such as rheumatoid arthritis and lupus (James et al., 2007). IBMX inhibits phosphodiesterase similar to Sildenafil (an FDA-approved drug). Hence, that IBMX can eliminate HCQ bradycardia effect can be immediately translated to a clinical trial. Thus, clinical translation of these findings may be beneficial to additional patient populations. Note that isoprotenalol or forskolin may induce response similar to those elicitied by IBMX. However, we chose to use IBMX because an FDA-approved drug with a similar mechanism exists.

### Limitations

The exact cardiac origin (i.e., ventricular, sinoatrial or atrial) of the hiPSC-CMs used in this study was not determined. Only cells that beat spontaneously at a rate similar to the human heart were selected for the experiments. The similarity between their response to HCQ or HCQ and IBMX compared to the responses of isolated SAN cells or SAN cells residing in the SAN tissue, suggest that they constitute a reliable and comparable model. Future use of SAN cells derived from hiPSC will clarify this point (Protze et al., 2016). Moreover, although Ca^2+^ and membrane clocks were discovered in hiPSCs (Zeevi-Levin et al., 2012), it remains to be determined whether they are coupled, and, if so, which signaling cascades connect them.

The concentrations of applied HCQ and IBMX were not consistent across the tested models. While a significant effect of 1 μM HCQ on beat interval was shown in single isolated SAN cells, a higher concentration was required to obtain a similar effect in hiPSC-CMs and SAN cells residing in SAN tissue. Similar trend was documented for IBMX. These results are not surprising when considering previous reports on the effects of other drugs on these experimental models (Janardhan et al., 2012; Ben-Ari et al., 2014). The lower sensitivity of drugs in SAN tissue compared to isolated cells may be related to diffusion limitations, specifically of drugs that affect internal signaling or which bind membrane channels from the cytosolic side. Lower sensitivity of hiPSC-CMs to drugs may be related to low densities of membrane channels such as I_f_ compared to rabbit SAN, and to different levels of cAMP/PKA activity. Future experiments are needed to further characterize these observations.

We predict here an indirect effect of HCQ on I_NCX_. Future experiments are needed to verify that it is an indirect effect of HCQ. It is challenging to measure I_NCX_ because its major contribution is during the non-linear phase of the action potential and performing steady voltage steps results in unphysiological activity. Performing ramp voltage steps is feasible, but the HCQ affects the non-linear slope of the action potential and thus even a ramp step will not be of physiological relevance. Moreover, based on our model observations (Supplementary Figure S6), voltage clamping would induce no significant measurable changes in I_NCX_ in the presence versus absence of HCQ.

## Data Availability

The original contributions presented in the study are included in the article/Supplementary Material, further inquiries can be directed to the corresponding author.

## Abbreviations

Ca_i_, intracellular Ca^2+^ concentration; Ca_jSR_, Ca^2+^ concentration at the junctional SR compartment; Ca_sub_, Ca^2+^ concentration at the subspace; HCQ, hydroxychloroquine; hiPSCs, human-induced pluripotent stem cells; hiPSC-CMs, human-induced pluripotent stem cell-derived cardiomyocytes; I_CaL_, L-type Ca^2+^ current; I_f_, funny current; I_Kr_, rapidly activating potassium current; I_NCX_, Na^+^-Ca^2+^ exchanger current; I_to_, transient outward potassium current; j_SRCarel_, flux of Ca^2+^ exiting the SR; IBMX, 3′-isobutylmethylxanthine; LCRs, local Ca^2+^ releases; SAN, sinoatrial node; SR, sarcoplasmic reticulum.
