# Supplementary material for: Increase in Ca2+-Activated cAMP/PKA Signaling Prevents Hydroxychloroquine-Induced Bradycardia of the Cardiac Pacemaker
Source: Front Physiol. 2022 May 11;13:839140. doi: 10.3389/fphys.2022.839140 (PMC9130770; doi:10.3389/fphys.2022.839140)
Supplement: Supplementary file 1 [file DataSheet1.docx]

Supplementary Material

# Supplementary Figures and Tables

|  | Control | HCQ 1 µM | HCQ 3 µM |
| --- | --- | --- | --- |
| Ca^2+^ transient parameters | | | |
| Beat interval [ms] | 518.27±30.58  (N=7) | 646.4±50.5**  (N=7) | 1403.44±318.81*  (N=6) |
| Beat interval SD [ms] | 53.85±12.31  (N=7) | 185.96±57.08*  (N=7) | 200.82±63.86*  (N=6) |
| Time to peak [ms] | 151.9±12.1  (N=7) | 182.55±19.07*  (N=7) | 219.09±42.22  (N=6) |
| Time to 50% relaxation [ms] | 241.32±20.3  (N=7) | 319.61±25.13**  (N=7) | 433.48±80.61*  (N=6) |
| Time to 90% relaxation [ms] | 356±18.76  (N=7) | 466.85±37.94**  (N=7) | 643.72±111.77*  (N=6) |
| Spontaneous diastolic LCR characteristics | | | |
| 50% spark duration [ms] | 31.03±0.43  (N=84) | 31.49±0.6  (N=47) | 31.29±0.66  (N=50) |
| Normalized amplitude [N.U] | 5.92±1.02  (N=84) | 3.65±0.59*  (N=47) | 1.06±0.06** ^##^  (N=29) |
| Amplitude difference [N.U] | 11.06±1.13  (N=84) | 32.35±4.33**  (N=43) | 23.48±1.05**  (N=50) |
| Spark length [µm] | 5.38±0.2  (N=84) | 4.42±0.2**  (N=47) | 4.29±0.18**  (N=50) |
| LCR period [ms] | 293.74±17.38  (N=77) | 544.9±57.54**  (N=43) | 818.87±63.64** ^##^  (N=47) |
| Number of LCR [1/sec*µm] | 0.06±0.01  (N=7) | 0.04±0.01  (N=7) | 0.08±0.03  (N=6) |
| Ca^2+^ signal of individual LCR (ms*µm*F/F_0_) | 806.6±92.7  (N=84) | 461.96±64.85**  (N=47) | 127.5±10.51** ^##^  (N=29) |
| LCR ensemble Ca^2+^ signal (ms*µm*F/F_0_/number of beat interval) | 80.35±42.67  (N=7) | 60.9±21.1  (N=7) | 49.4±16.9  (N=7) |

**Table S1.** The effects of 1 or 3 µM HCQ on Ca^2+^ transient and spontaneous LCR characteristics (N presents the number of cells (Ca^2+^ transient) or number of LCR events). * P<0.05 vs. control, ** p<0.01 vs. control, ^#^ p<0.05 vs. HCQ 1 µM and ^##^ p<0.01 vs. HCQ 1µM.

|  | Control  (N=18) | HCQ 10 µM  (N=18) | HCQ 10 µM+  IBMX 50 µM  (N=6) |
| --- | --- | --- | --- |
| Ca^2+^ transient | | | |
| Beat interval [ms] | 1309.3 ± 97.7 | 2029.6 ± 239.6 ** | 1301.8 ± 79.6 ^#^ |
| Beat interval SD [ms] | 128 ± 20.7 | 201.4 ± 35.2 * | 159.7 ± 37.7 |
| Time to peak [ms] | 316.5 ± 26.8 | 430.7 ± 39.7 ** | 317.9 ± 18.5 ^#^ |
| Time to 50% relaxation [ms] | 487.3 ± 32.8 | 667.4 ± 53.4 ** | 520.8 ± 23.7 ^#^ |
| Time to 90% relaxation [ms] | 663.9 ± 36.6 | 976 ± 71 ** | 764.7 ± 41.2 |

**Table S2.** The effects of 10 µM HCQ and 10 µM HCQ + 50 µM IBMX on Ca^2+^ transient (N presents the number of cells). * p<0.05 vs. control, ** p<0.01 vs. control, ^#^ p<0.05 vs. 10 µM HCQ.

|  | Control  (N=23) | HCQ 10 µM  (N=23) | HCQ 10 µM + IBMX 10µM  (N=13) | HCQ 10 µM + IBMX 50 µM  (N=10) |
| --- | --- | --- | --- | --- |
| Ca^2+^ transients | | | | |
| Beat interval [ms] | 468.6±37.2 | 863.6±85 ** | 693.8±68.7 ** | 393.8±25.1 ^##^ |
| Beat interval SD [ms] | 123.9±23.6 | 242.9±42.9 * | 230.3±46.3 * | 86.8±22.7 ^##^ |
| Time to peak [ms] | 108.3±8.8 | 151.6±30.2 | 150.4±29.3 | 106.2±12 |
| Time to 50% relaxation [ms] | 173.9±15.5 | 217.5±35.3 | 192±32.8 | 164±21.1 |
| Time to 90% relaxation [ms] | 286.8±32 | 308.7±54.3 | 266±46.7 | 218.9±27.4 |

**Table S3.** The effects of 10 µM HCQ and 10 µM HCQ + 10 µM IBMX on Ca^2+^ transient (N presents the number of cells). * p<0.05 vs. control, ** p<0.01 vs. control, ^##^ p<0.01 vs. 10 µM HCQ.

|  | Control | HCQ 1 µM | HCQ 1 µM+  IBMX 10 µM |
| --- | --- | --- | --- |
| Ca^2+^ transient parameters | | | |
| Beat interval [ms] | 518.27±30.58  (N=7) | 678.04±63.99*  (N=7) | 442.45±21.32^#^  (N=7) |
| Beat interval SD [ms] | 53.85±12.31  (N=7) | 170.72±44.65*  (N=7) | 113.86±26.6  (N=7) |
| Time to peak [ms] | 151.9±12.1  (N=7) | 159.04±28.18  (N=7) | 137.25±13.15  (N=7) |
| Time to 50% relaxation [ms] | 241.32±20.3  (N=7) | 245.25±30.49  (N=7) | 216.15±20.77  (N=7) |
| Time to 90% relaxation [ms] | 356±18.76  (N=7) | 387.43±32.86  (N=7) | 303.37±18.87^##^  (N=7) |
| Spontaneous diastolic LCR Characteristics | | | |
| 50% spark duration [ms] | 31.03±0.43  (N=84) | 29.84±0.33*  (N=84) | 29.47±0.33**  (N=70) |
| Normalized amplitude [N.U] | 5.92±1.02  (N=84) | 3.65±0.59*  (N=47) | 4.71±0.92  (N=70) |
| Amplitude difference [N.U] | 11.06±1.13  (N=84) | 2.88±2.48**  (N=82) | 8.98±1.78^#^  (N=66) |
| Spark length [µm] | 5.38±0.2  (N=84) | 4.79±0.17*  (N=84) | 5±0.2  (N=70) |
| LCR period [ms] | 293.74±17.38  (N=77) | 433.42±30.01**  (N=79) | 303.32±18.92^##^  (N=65) |
| Number of LCR [1/sec*µm] | 0.06±0.01  (N=7) | 0.04±0.01  (N=7) | 0.12±0.03  (N=7) |
| Ca^2+^ signal of individual LCR (ms*µm*F/F0) | 806.66±92.67  (N=89) | 461.96±64.85**  (N=47) | 597.57±101.41^##^  (N=70) |
| LCR ensemble Ca^2+^ signal (ms*µm*F/F_0_/number of beat interval) | 80.35±42.67  (N=7) | 60.9±21.1  (N=7) | 72.9±32.9  (N=7) |

**Table S4.** The effects of 1 µM HCQ or 1 µM HCQ and 10 µM IBMX on Ca^2+^ transient and spontaneous LCR characteristics (N presents the number of cells (Ca^2+^ transient) or number of LCR events). * P<0.05 vs. control, ** p<0.01 vs. control, ^#^ p<0.05 vs. HCQ 1 µM and ^##^ p<0.01 vs. HCQ 1 µM.


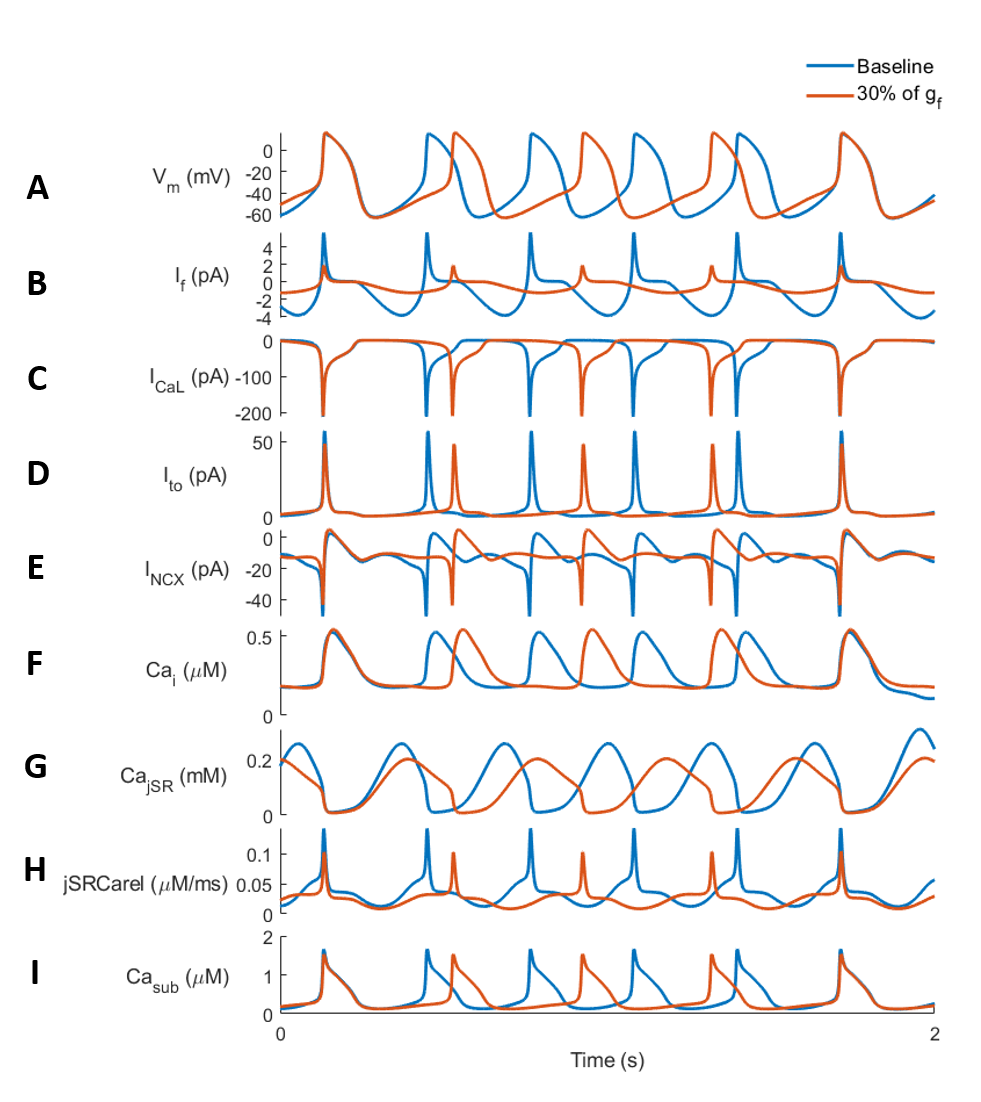


**Figure S1: Major currents and Ca^2+^ cycling in simulated rabbit sinoatrial node cells in response to decrease in I_f_ conductance.** Coupled-clock function of control (blue) the response to only decrease in HCN (funny) current (I_f_) (red) density by 70%. (A) Membrane voltage (V_m_) and representative beat intervals, (B) I_f_, (C) L-type Ca^2+^ current (I_CaL_), (D) transient potassium current (I_t0_), (E) Na^+^-Ca^2+^ exchanger current (I_NCX_), (F) intracellular Ca^2+^ concentration (Ca_i_), (G) Ca^2+^ concentration in the junctional SR compartment (Ca_jSR_), (H) flux of Ca^2+^ exiting the SR (j_SRCarel_) and (I) Ca^2+^ concentration in the subspace (Ca_sub_).

**
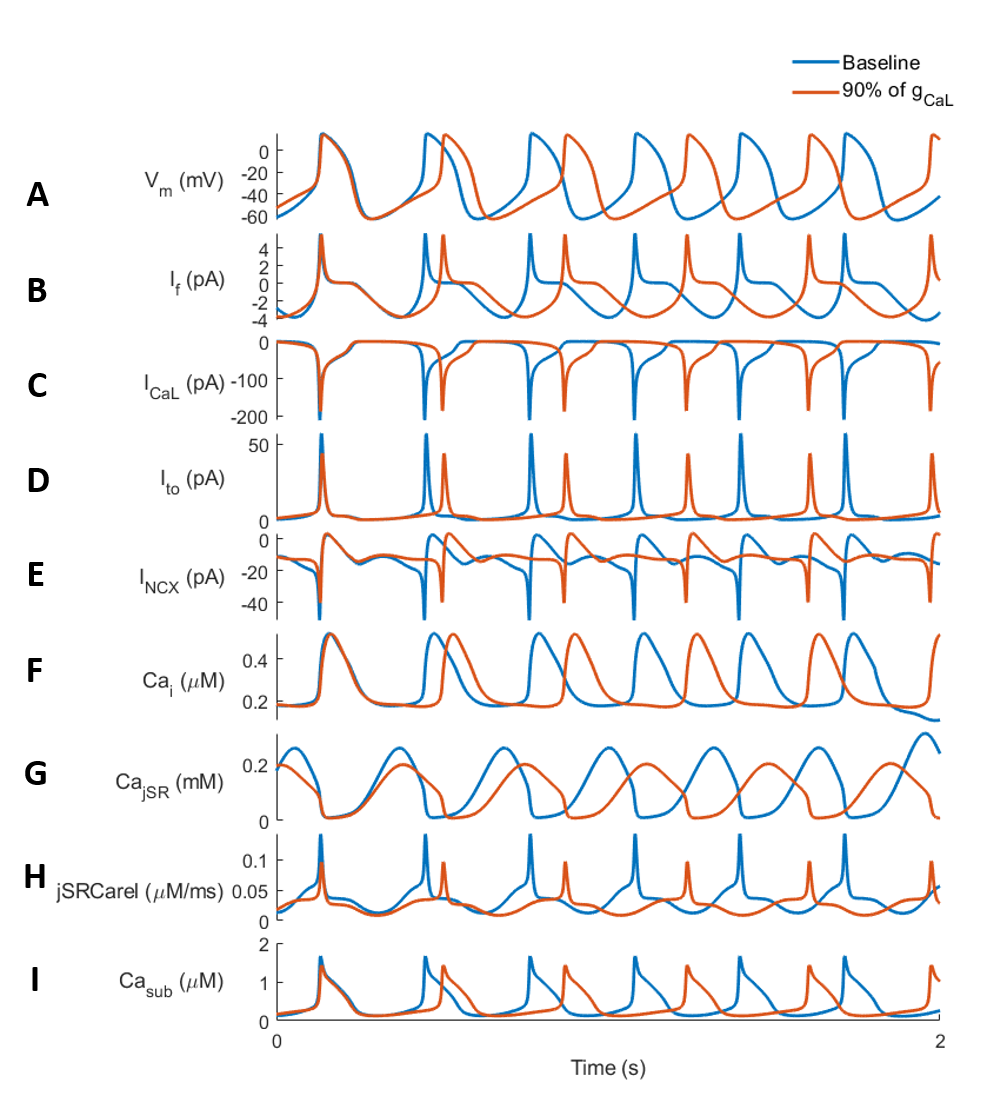
**

**Figure S2: Major currents and Ca^2+^ cycling in simulated rabbit sinoatrial node cells in response to decrease in I_CaL_** **conductance.** Coupled-clock function of control (blue) the response to only decrease in L-type Ca^2+^ current (I_Ca,_L) conductance (red) by 10%. (A) Membrane voltage (V_m_) and representative beat intervals, (B) I_f_, (C) I_CaL_, (D) transient potassium current (I_t0_), (E) Na^+^-Ca^2+^ exchanger current (I_NCX_), (F) intracellular Ca^2+^ concentration (Ca_i_), (G) Ca^2+^ concentration in the junctional SR compartment (Ca_jSR_), (H) flux of Ca^2+^ exiting the SR (j_SRCarel_) and (I) Ca^2+^ concentration in the subspace (Ca_sub_).


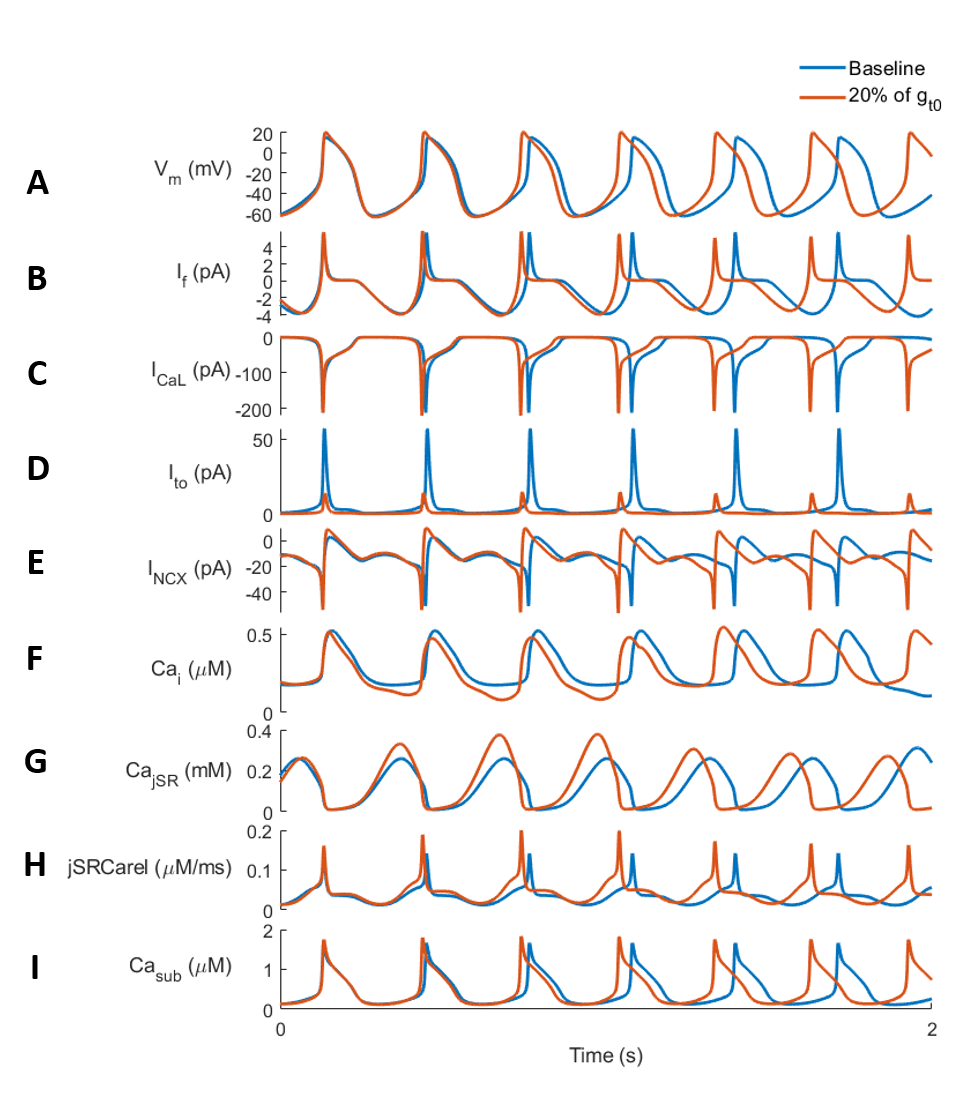


**Figure S3: Major currents and Ca^2+^ cycling in simulated rabbit sinoatrial node cells in response to decrease in I_t0_ conductance.** Coupled-clock function of control (blue) the response to only decrease in transient potassium current (I_t0_) conductance by 80%. (A) Membrane voltage (V_m_) and representative beat intervals, (B) I_f_, (C) I_CaL_, (D) I_t0_, (E) Na^+^-Ca^2+^ exchanger current (I_NCX_), (F) intracellular Ca^2+^ concentration (Ca_i_), (G) Ca^2+^ concentration in the junctional SR compartment (Ca_jSR_), (H) flux of Ca^2+^ exiting the SR (j_SRCarel_) and (I) Ca^2+^ concentration in the subspace (Ca_sub_).

**
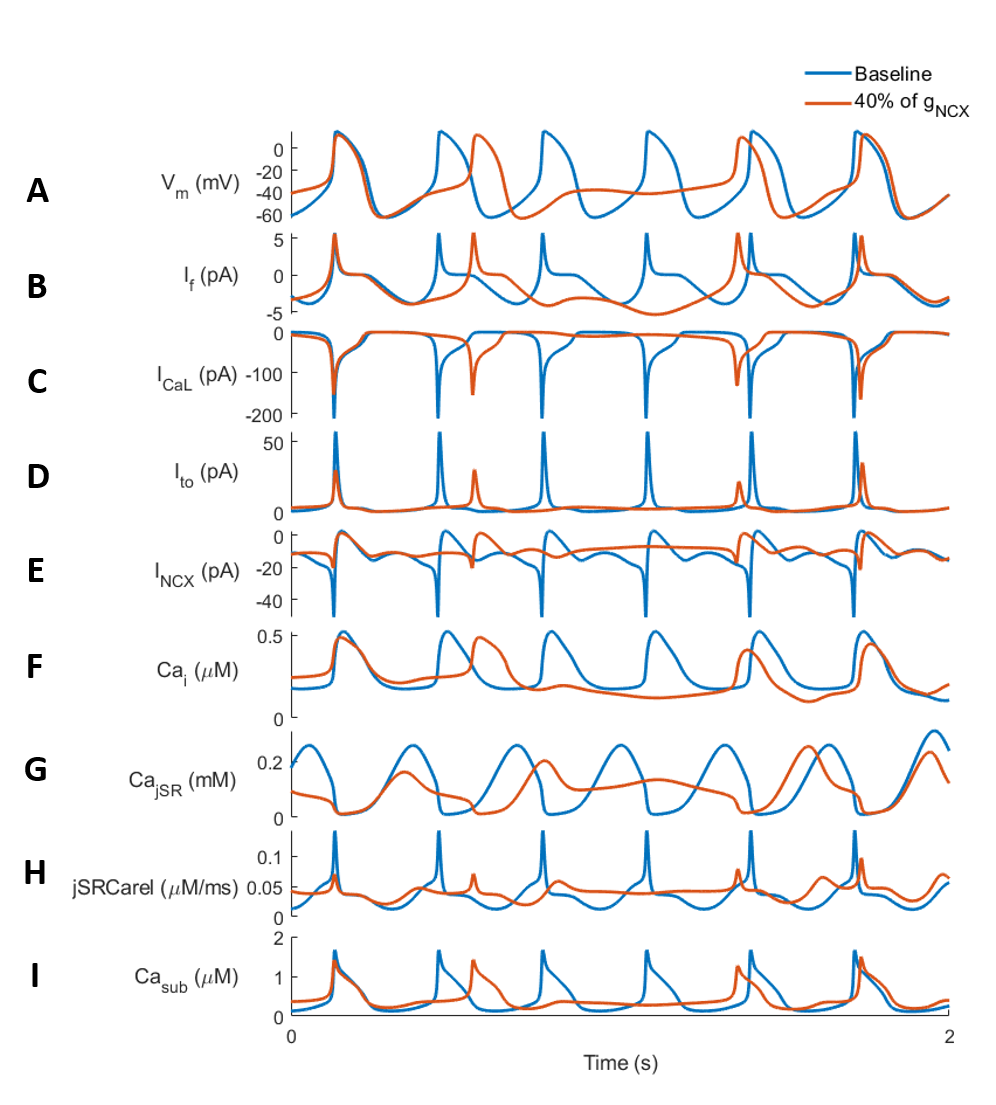
**

**Figure S4: Major currents and Ca^2+^ cycling in simulated rabbit sinoatrial node cells in response to decrease in I_NCX_ conductance.** Coupled-clock function of control (blue) the response to only decrease in transient potassium current (I_t0_) conductance by 80%. (A) Membrane voltage (V_m_) and representative beat intervals, (B) I_f_, (C) I_CaL_, (D) I_t0_, (E) Na^+^-Ca^2+^ exchanger current (I_NCX_), (F) intracellular Ca^2+^ concentration (Ca_i_), (G) Ca^2+^ concentration in the junctional SR compartment (Ca_jSR_), (H) flux of Ca^2+^ exiting the SR (j_SRCarel_) and (I) Ca^2+^ concentration in the subspace (Ca_sub_).

**
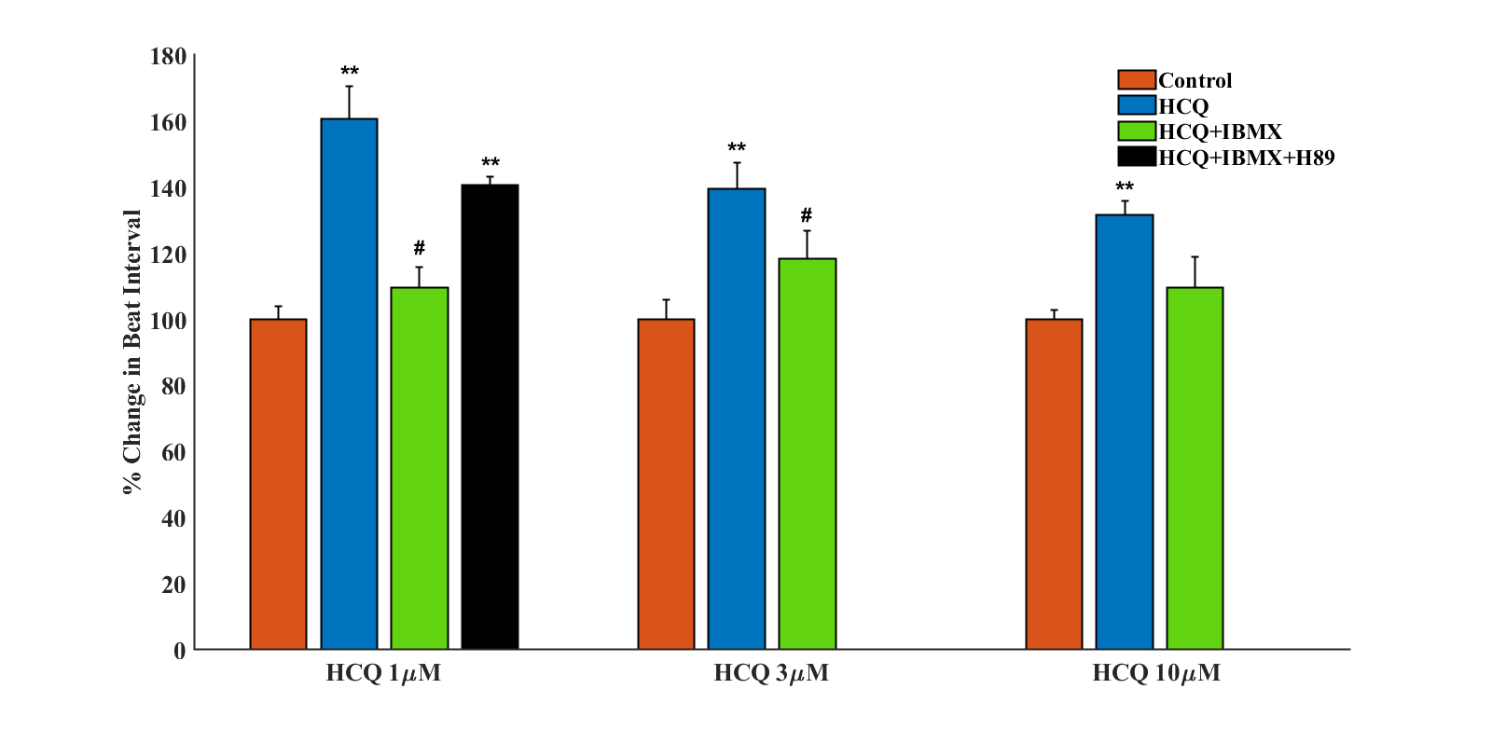
**

**Figure S5: The effect of hydroxychloroquine (HCQ) with 3’-isobutylmethylxanthine** (**IBMX) on single rabbit sinoatrial node cells (SAN).** Percentage of change in beat interval in control (red, n=6) rabbit SAN cells treated with 1 µM (n=6), 3 µM (n=6) and 10 µM (n=6) HCQ (blue) + 10 µM IBMX-treated single rabbit sinoatrial node (SAN) cells (green, n=6), respectively. A subset SAN cells that treated with 1µM HCQ+10 µM IBMX were also exposed to 10 µM (black, n=11). * p<0.05 vs. control, ** p<0.01 vs. control,  ^#^ p<0.05 vs. HCQ 1µM.


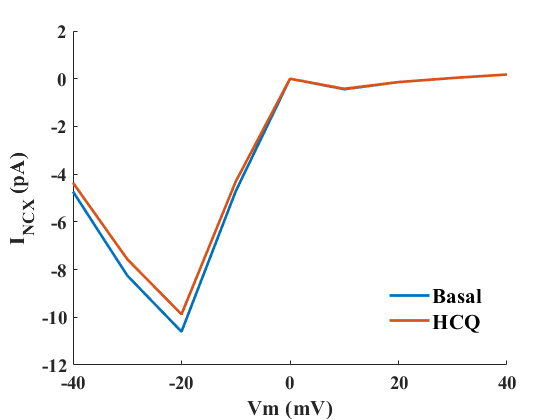


**Figure S6. Computational simulation of I_NCX_ with and without 1 µM HCQ in response to voltage clamp protocol.**
